# Commentary: Motor Imagery during Action Observation: A Brief Review of Evidence, Theory and Future Research Opportunities

**DOI:** 10.3389/fnhum.2017.00025

**Published:** 2017-01-25

**Authors:** Howie J. Carson, Dave J. Collins

**Affiliations:** Institute for Coaching and Performance, School of Sport and Wellbeing, University of Central LancashirePreston, UK

**Keywords:** motor Imagery, motor control, sport psychology, observational learning, coaching psychology

Eaves et al. (2016) recently provided an informative review concerning motor imagery *during* action observation. Specifically, they addressed its effectiveness, mechanisms, and future directions for study. While there is an apparent scarcity of studies to date, it is clear that much similarity exists in neural activity during action, observation, and imagery, with clear *super-additive* advantages for performing the latter two concurrently. We could not agree more with these authors when it comes to encouraging practitioners (from our perspective, sport psychologists and coaches) to deploy mental simulation interventions as central pillars to assist a range of motor skill challenges (e.g., executing under high competitive anxiety, skill acquisition). Notably, the review also provided coverage of motoric factors (Carson and Collins, 2016) to offer an integrated understanding, something that has been somewhat neglected in other areas of psychology (e.g., the anxiety–performance relationship; Cheng et al., 2009). Reflecting this motoric emphasis, there are a number of interesting links, possibilities, and research directions which accrue from this paper. As Eaves et al. explain, “AO [action observation] evokes an internal representation of the observed movement” (p. 1). Accordingly, we raise several issues requiring attention from the translational research literature (Christina, 1987) and extend some of the suggestions by Eaves et al. Understandably, article length restrictions may have impacted on Eaves et al. in providing this necessary detail that we feel warrants additional mention. Therefore, it is not our intention to take away from the quality work presented but rather, to support its desired aims.

Firstly, the issue of task difficulty in relation to the nature (i.e., content and automaticity) of motoric structures over which the observed and imagined information act. Eaves et al. (2016) discuss a *dual-action* simulation framework for conceptualizing previous empirical findings. However, such dual-action effects might vary for tasks internalized in different ways, for instance rhythmical window wiping (highly internalized) might show different dual-actions than a complex high jump skill (less well-internalized). Toner and Moran (2015) discuss the advantage of high-level athletes purposefully *not* committing their skills to totally automatic control as a strategy to ensure scope for future adaptability. Indeed, applied research from the multi-action plan (MAP) perspective supports this notion. Data show that optimal performances are achieved by elite-level athletes through both automatic *and* controlled execution processes (Bortoli et al., 2012), whereby consciously controlled motor processing can positively assist performances under psychological pressure and physical fatigue. Distinction between these two optimal execution modes and their relative processing efficiency, therefore, offers complexity regarding the translation of fundamental, theory-driven research for use with populations such as athletes. When a task is so extremely familiar and simple (with limited degrees of freedom involved; e.g., finger pinching) it is possible that the observer quickly extracts sufficient relevant information from the model through retinal-dependent feedback (or *attention*), then switches to a state of *intention* whereby the motor representation is generated and retrieved through imagery processes (Wertheim, 1981; Loze et al., 1999). Of course, this would only be possible with a high degree of efficiency *if* the representation was easily accessible *and* well-established within long-term memory. If the skill is less well-established (perhaps due to its complexity), it might be that the interplay is different between observing and then imaging (e.g., watch–image–watch–image/watch; cf. Smith et al., 1998).

As a related but distinct issue, it may also be beneficial to consider the observation-based review of errors, and what associated processes can bring to the learning process. For example, the generation of efference copy (what the movement should feel like) which learners' can then use for intentive comparison and subsequent, enhanced learning (cf. Gallagher, 2000). Of relevance, there is clear evidence that similar neural mechanisms are involved in error detection for one's own movements or when observing others (van Schie et al., 2004). Whether the case is of a well-known or being-learnt task, however, for observation and imagery to be optimized within the applied sport setting, a better understanding of these processes across complex moves are required.

A second issue relates to what the observer focusses on during the observation and/or imagery process. This is likely important because studies have demonstrated different learning/performance effects when an athlete adopts different attentional foci. In javelin throwing, MacPherson et al. (2008) found that focusing on holistic-rhythm resulted in a more consistent pattern of variability across kinematics whereas focusing on a single element (e.g., fast arm) lowered the variability of that component and increased the variability across others; thus disrupting the balance of control. Focusing on “core components” of an action (those that are essential for optimal performance), as advocated by the MAP, has been shown to result in optimal performance levels even though they are underpinned by an inefficient (according to the neural efficiency hypothesis; Hatfield and Kerick, 2007), dissimilar state of neural assemblies (desynchronized theta/alpha power) when compared to automatic processing (Bertollo et al., 2016). A key question here, then, is whether the nature of what is imaged whilst observing changes the extent to which motor regions are activated in the brain and thus, the representation formed (cf. van Schie et al., 2004; Neuper et al., 2009). Given that this content will change with experience, the case of skill refinement (adjusting an already established skill; Carson and Collins, 2011) is a particular case for further consideration. As one of many issues, how will the pattern of imagery/observation change when moving from well-established but suboptimum components to replace them with new but comparatively unknown elements.

Finally, there has been growing evidence to support the beneficial impact on mental imagery when employing PETTLEP (Holmes and Collins, 2001) principles. On the basis that observation and imagery processes share, at the very least, similar neural pathways, could the benefits already shown by combining imagery with observation be improved even further by attention to these important elements? We suggest that examination of such effects would be welcomed and well-situated to inform other more prominent areas of research within motor control; namely, that concerning an athlete's focus of attention (Wulf, 2013).

## Author contributions

HC and DC contributed equally to the ideas and writing of the article.

### Conflict of interest statement

The authors declare that the research was conducted in the absence of any commercial or financial relationships that could be construed as a potential conflict of interest.

## References

[B1] Bertollo M. di Fronso S. Filho E. Conforto S. Schmid M. Bortoli L. (2016). Proficient brain for optimal performance: the MAP model perspective. PeerJ 4:e2082 10.7717/peerj.2082 27257557 PMC4888308

[B2] Bortoli L. Bertollo M. Hanin Y. Robazza C. (2012). Striving for excellence: a multi-action plan intervention model for shooters. Psychol. Sport Exerc. 13, 693–701. 10.1016/j.psychsport.2012.04.006

[B3] Carson H. J. Collins D. (2011). Refining and regaining skills in fixation/diversification stage performers: the five-a model. Int. Rev. Sport Exerc. Psychol. 4, 146–167. 10.1080/1750984X.2011.613682

[B4] Carson H. J. Collins D. (2016). The fourth dimension: a motoric perspective on the anxiety–performance relationship. Int. Rev. Sport Exerc. Psychol. 9, 1–21. 10.1080/1750984X.2015.1072231 26692896 PMC4662095

[B5] Cheng W.-N. K. Hardy L. Markland D. (2009). Toward a three-dimensional conceptualization of performance anxiety: rationale and initial measurement development. Psychol. Sport Exerc. 10, 271–278. 10.1016/j.psychsport.2008.08.001

[B6] Christina R. W. (1987). Motor learning: Future lines of research, in The Cutting Edge in Physical Education and Exercise Science Research, eds Safrit M. J. Eckert H. M. (Champaign, IL: Human Kinetics), 26–41.

[B7] Eaves D. L. Riach M. Holmes P. S. Wright D. J. (2016). Motor imagery during action observation: a brief review of evidence, theory and future research opportunities. Front. Neurosci. 10:514. 10.3389/fnins.2016.00514 27917103 PMC5116576

[B8] Gallagher S. (2000). Philosophical conceptions of the self: implications for cognitive science. Trends Cogn. Sci. 4, 14–21. 10.1016/S1364-6613(99)01417-5 10637618

[B9] Hatfield B. D. Kerick S. E. (2007). The psychology of superior sport performance: A cognitive and affective neuroscience perspective, in Handbook of Sport Psychology, 3rd Edn., eds Eklund R. C. Tenenbaum G. (Hoboken, NJ: John Wiley & Sons Inc), 84–109.

[B10] Holmes P. S. Collins D. J. (2001). The PETTLEP approach to motor imagery: a functional equivalence model for sport psychologists. J. Appl. Sport Psychol. 13, 60–83. 10.1080/10413200109339004

[B11] Loze G. M. Collins D. Shaw J. C. (1999). EEG alpha rhythm, intention and oculomotor control. Int. J. Psychophysiol. 33, 163–167. 10.1016/S0167-8760(99)00024-0 10489080

[B12] MacPherson A. C. Collins D. Morriss C. (2008). Is what you think what you get? Optimizing mental focus for technical performance. Sport Psychol. 22, 288–303. 10.1123/tsp.22.3.288

[B13] Neuper C. Scherer R. Wriessnegger S. Pfurtscheller G. (2009). Motor imagery and action observation: modulation of sensorimotor brain rhythms during mental control of a brain-computer interface. Clin. Neurophysiol. 120, 239–247. 10.1016/j.clinph.2008.11.015 19121977

[B14] Smith D. Collins D. Hale B. (1998). Imagery perspectives and Karate performance. J. Sport Sci. 16, 103–104.

[B15] Toner J. Moran A. (2015). Enhancing performance proficiency at the expert level: considering the role of ‘somaesthetic awareness’. Psychol. Sport Exerc. 16, 110–117. 10.1016/j.psychsport.2014.07.006

[B16] van Schie H. T. Mars R. B. Coles M. G. H. Bekkering H. (2004). Modulation of activity in medial frontal and motor cortices during error observation. Nat. Neurosci. 7, 549–554. 10.1038/nn1239 15107858

[B17] Wertheim A. H. (1981). Occipital alpha activity as a measure of retinal involvement in oculomotor control. Psychophysiology 18, 432–439. 10.1111/j.1469-8986.1981.tb02476.x 7267926

[B18] Wulf G. (2013). Attentional focus and motor learning: a review of 15 years. Int. Rev. Sport Exerc. Psychol. 6, 77–104. 10.1080/1750984X.2012.723728

